# Antioxidant, antimicrobial, antiparasitic, and cytotoxic properties of various Brazilian propolis extracts

**DOI:** 10.1371/journal.pone.0172585

**Published:** 2017-03-30

**Authors:** Rejane Pina Dantas Silva, Bruna Aparecida Souza Machado, Gabriele de Abreu Barreto, Samantha Serra Costa, Luciana Nalone Andrade, Ricardo Guimarães Amaral, Adriana Andrade Carvalho, Francine Ferreira Padilha, Josiane Dantas Viana Barbosa, Marcelo Andres Umsza-Guez

**Affiliations:** 1 Department of Pharmacy, Federal University of Bahia, Salvador, Bahia, Brazil; 2 Department of Biotechnology and Food, Faculty of Technology, SENAI/CIMATEC, National Service of Industrial Learning – SENAI, Salvador, Bahia, Brazil; 3 Institute of Technology in Health, Faculty of Technology, SENAI/CIMATEC, National Service of Industrial Learning – SENAI, Salvador, Bahia, Brazil; 4 Department of Physiology, Federal University of Sergipe, São Cristovão, Sergipe, Brazil; 5 Department of Pharmacy, Federal University of Sergipe, Lagarto, Sergipe, Brazil; 6 Institute of Research and Technology, Tiradentes University, Aracaju, Sergipe, Brazil; 7 Department of Biotechnology, Federal University of Bahia, Salvador, Bahia, Brazil; Tallinn University of Technology, ESTONIA

## Abstract

Propolis is known for its biological properties and its preparations have been continuously investigated in an attempt to solve the problem of their standardization, an issue that limits the use of propolis in food and pharmaceutical industries. The aim of this study was to evaluate *in vitro* antioxidant, antimicrobial, antiparasitic, and cytotoxic effects of extracts of red, green, and brown propolis from different regions of Brazil, obtained by ethanolic and supercritical extraction methods. We found that propolis extracts obtained by both these methods showed concentration-dependent antioxidant activity. The extracts obtained by ethanolic extraction showed higher antioxidant activity than that shown by the extracts obtained by supercritical extraction. Ethanolic extracts of red propolis exhibited up to 98% of the maximum antioxidant activity at the highest extract concentration. Red propolis extracts obtained by ethanolic and supercritical methods showed the highest levels of antimicrobial activity against several bacteria. Most extracts demonstrated antimicrobial activity against *Staphylococcus aureus*. None of the extracts analyzed showed activity against *Escherichia coli* or *Candida albicans*. An inhibitory effect of all tested ethanolic extracts on the growth of *Trypanosoma cruzi* Y strain epimastigotes was observed in the first 24 h. However, after 96 h, a persistent inhibitory effect was detected only for red propolis samples. Only ethanolic extracts of red propolis samples R01Et.B2 and R02Et.B2 showed a cytotoxic effect against all four cancer cell lines tested (HL-60, HCT-116, OVCAR-8, and SF-295), indicating that red propolis extracts have great cytotoxic potential. The biological effects of ethanolic extracts of red propolis revealed in the present study suggest that red propolis can be a potential alternative therapeutic treatment against Chagas disease and some types of cancer, although high activity of red propolis *in vitro* needs to be confirmed by future *in vivo* investigations.

## Introduction

The use of propolis, a resinous substance collected by honeybees, for skin treatment and healing of wounds and ulcers has been reported since ancient times [1]. However, detailed studies of propolis constituents and their biological properties have been carried out only in recent decades [2].

Due to the great biodiversity of Brazil, propolis composition in different geographic regions varies, and several distinct propolis types have been described in this country [3, 4]. Currently, 13 different groups of propolis can be distinguished in Brazil. Propolis group 13, recently described in the northeastern region, is characterized by a strong red color and some other characteristics that set it apart from other propolis groups found in Brazil [5, 6]. In 2012, red propolis from Alagoas and its extract were given a certificate of Geographical Indication by the Brazilian National Institute of Industrial Property [7].

Propolis has been extensively investigated, because its constituents exhibit several properties of interest for the scientific community. Biological effects of propolis have been described mainly in relation to propolis antioxidant activity [8–10], antimicrobial effects, and cytotoxicity [11–15]. Additionally, an increasing number of studies are being performed to investigate antiparasitic activity of propolis [16–18].

The chemical composition of propolis depends on various factors, such as its botanical origin, geographical origin, and collection time [19, 20]. Bankova [19], indicated that the process of standardization of propolis preparations is efficient when it is based on the classification according to the plant source used by the bees, but there is still need for further research to achieve reliable classification. The standardization problem limits the application of propolis in food and pharmaceutical industries [12].

Propolis extracts are obtained by different extraction methods. The conventional technique uses ethanol as the extraction solvent, whereas alternative methods, such as supercritical fluid extraction, have also been described [21]. It is known that extraction method also influences the obtained extract, and different extracts from the same propolis sample may exhibit dissimilar properties. The yield and selectivity for some compounds are directly affected by the extraction method [22–24].

The aim of this study was to perform *in vitro* evaluation of antioxidant, antimicrobial, antiparasitic, and cytotoxic properties of extracts of red, green, and brown propolis from different regions of Brazil obtained by ethanolic and supercritical extraction methods.

## Material and methods

### Reagents

Ethanol (HPLC grade) was obtained from Merck Co. (Darmstadt, Germany). Doxorubicin (>98% purity), resazurin, and potassium persulfate were obtained from Sigma-Aldrich Chemical Co. (St. Louis, MO, USA). Carbon dioxide (CO_2_; 99.9% purity) was purchased from White Martins Gases Industrials (São Paulo, Brazil). 2,2′-Azinobis-(3-ethylbenzothiazoline-6-sulfonic) acid (ABTS) and (±)-6-hydroxy-2,5,7,8-tetramethylchromane-2-carboxylic acid (Trolox) were acquired from Sigma-Aldrich Chemical Co. (St. Louis, MO, USA).

### Sample preparation

Propolis samples were donated by Apis Nativa Produtos Naturais LTDA Company. These samples were obtained from the same geographical regions as the samples examined by our group in a previous study [25]. However, the samples used in this study were obtained in a different year (2014). Consequently, they were from a batch (B2) different from that of the samples analyzed previously.

Red propolis samples R01.B2 (lot number L02, red color, Sergipe apiary, Brazil, resinous appearance) and R02.B2 (lot number L01, red color, Alagoas apiary, Brazil, resinous appearance) were collected in the northeastern part of Brazil, in mangrove regions. Green samples G01.B2 (lot number L 66(4), green color, Minas Gerais apiary, Brazil, resinous appearance) and G02.B2 (lot number L 66(3), green color, Minas Gerais apiary, Brazil, resinous appearance) were collected in the southeastern region and another sample of green propolis (lot number L 103, green color, Paraná apiary, Brazil, resinous appearance) was collected in the southern region of Brazil. All brown propolis samples were collected in the southern region of Brazil, B01.B2 (lot number L 76, brown color, Santa Catarina apiary, Brazil, resinous appearance), B02.B2 (lot number L 73(3), brown color, Rio Grande do Sul apiary, Brazil, resinous appearance) and B03.B2 (lot number L 02, brown color, Paraná apiary, Brazil, resinous appearance).

Before use, raw propolis was ground to a granulated powder using a food grinder. After grinding, propolis samples were stored in a freezer until use. Table 1 below provides descriptions of propolis samples. Table 1 below shows the sample propolis identification.

**Table 1:** Propolis sample identification by the color, region, and state of origin.

Identification	Color	Region	State of Origin
R01.B2	Red	Northeast	Sergipe
R02.B2	Red	Northeast	Alagoas
G01.B2	Green	Southeast	Minas Gerais
G02.B2	Green	Southeast	Minas Gerais
G03.B2	Green	South	Paraná
B01.B2	Brown	South	Santa Catarina
B02.B2	Brown	South	Rio Grande do Sul
B03.B2	Brown	South	Paraná

### Ethanolic extract production

Ground propolis (2 g) was extracted with ethanol (15 mL, 80%) by mixing the samples for 30 min under constant agitation (Incubation Shaker MA 420/MARCONI—Brazil) at 70°C and 710 rpm. The extract was recovered by centrifugation (Centrifuge SIGMA 2–16 KL) for 11 min at 8,800 rpm and 5°C. Then, an additional centrifugation step was performed with 10 mL of ethanol (80%). The supernatant was collected, homogenized, and kept at 50°C until completely dry. Afterwards, the extracts were stored in tubes, wrapped in aluminum foil at inert atmospheric conditions (N_2_) to avoid degradation. All extracts were kept at 5°C until use [6].

### Supercritical extract production

To obtain propolis extracts by supercritical extraction, a Supercritical Fluid Extractor SFT-110 (Supercritical Fluid Technologies, Inc.) was used. In each experiment, the extraction cell comprised 7.5 g of ground propolis sample with 1% ethanol as co-solvent (m/m), wool, and glass pearls. The extraction conditions were as follows: pressure, 350 bar; temperature, 50°C; co-solvent, 1% ethanol (m/m); CO_2_ flow, 6 g·min^-1^. The extraction time was about 2.5 h [22]. The extracts collected in a vial were stored wrapped in aluminum foil at inert atmospheric conditions (N_2_) to avoid degradation. The extracts were kept at 5°C until use [6, 22].

### Determination of *in vitro* antioxidant activity

Antioxidant activity was determined by using the ABTS method according to Van der Berg et al. approach [26] with modifications described by Kim et al. [27]. First, a 7 mM ABTS solution was prepared in distilled water. A 5-mL aliquot of this solution was removed and mixed with 88 μL of a 2.45 mM potassium persulfate solution to produce ABTS^•+^ radical. The final product was incubated for 16 h in dark conditions in order to enable production of ABTS^•+^ radical cation. ABTS^•+^ radical solution was then diluted in ethanol to the absorbance value of 0.7 ± 0.05 as determined at 734 nm.

The extract samples were diluted to concentrations of 0.1 mg.ml^-1^, 0.5 mg.ml^-1^ and 0.75 mg.ml^-1^. The extract samples were diluted to concentrations of 0.1 mg∙mL^-1^, 0.5 mg∙mL^-1^, and 0.75 mg∙mL^-1^. In a dark place, a 20-μL aliquot of each sample was transferred to a test tube containing 2 mL of ABTS•+ final solution. After a 6-min incubation, the absorbance of the samples was determined at 734 nm. The results were expressed in Trolox equivalent antioxidant capacity values.

### Antimicrobial activity

The gram-positive bacteria *Staphylococcus aureus* (ATCC 25923) and *Enterococcus* sp. (ATCC 29712), gram-negative bacteria *Klebsiella* sp. (ATCC 1706 / 700603), *Escherichia coli* (ATCC 25922), and pathogen fungus *Candida albicans* (ATCC 18804) were used for antimicrobial tests. The strains were supplied by the Bacterial Culture Collection of the Instituto Oswaldo Cruz—FIOCRUZ (Manguinhos, Rio de Janeiro, Brazil).

Bacteria were activated in liquid brain-heart infusion medium (BHI) (Sigma-Aldrich Chemical Co.—St. Louis, MO, USA) at 37°C for 24 h. They were grown in BHI agar plates for inoculum preparation and adjusted to 0.5 McFarland standard scale turbidity equivalent at a concentration of 1.0×10^8^ CFU/mL. Tenfold serial dilutions of the broth were performed to yield suspensions containing 1.0×10^4^ CFU/mL (pre-inoculum), which were used in the tests. The fungal inoculum was determined from a subculture on Potato Dextrose Agar (PDA—Sigma-Aldrich Chemical Co.—St. Louis, MO, USA) for 24 h at 35°C in order to ensure its purity and viability. Then, five strains of the fungal culture were suspended in sterile saline solution and vortexed for 15 s. The concentration of cells was adjusted with RPMI 1640 medium to a standard liquid suspension containing 1.0×10^3^ CFU/mL.

The minimum inhibitory concentrations (MICs) of ethanolic and supercritical extracts were determined by the 96-well plate microdilution method established at the Clinical and Laboratory Standards Institute [28, 29]. During MIC determination, the initial bacterial inoculum contained 1.0×10^4^ CFU/mL, whereas the fungal inoculum contained 1.0×10^3^ CFU/mL. Extract concentrations were in the range from 1000 to 31.3 μg∙mL^-1^. Resazurin (0.01% m/v) (Sigma, ST. Louis, MO, USA) was used to assess viability of the microorganisms. MIC was defined as the lowest concentration inhibiting bacterial growth (without visible growth).

### In vitro activity of ethanolic propolis extracts against Trypanosoma cruzi (Y strain) epimastigotes

Assays were performed on epimastigotes of *T*. *cruzi* Y strain donated by FIOCRUZ (Salvador, Bahia, Brazil). Epimastigotes were cultivated in liver infusion tryptose medium supplemented with 10% fetal bovine serum (Sigma-Aldrich), 1% hemin, and 1% R9 (Sigma-Aldrich) at 26°C and harvested during the exponential phase of growth. Then, the cell suspension was centrifuged at 2,500 rpm for 12 min until parasites reached a cell density of 3×10^6^ epimastigotes/mL. Susceptibility of *T*. *cruzi* to propolis was assessed at extract concentrations of 75 mg∙mL^-1^ and 300 mg∙mL^-1^. Assay plates were subsequently incubated at 26°C. After 24 h and 96 h, epimastigotes that remained alive were counted using a Neubauer chamber [30, 31].

### *In vitro* cytotoxicity

Cytotoxicity of propolis extracts against four human tumor cell lines, OVCAR-8 (ovarian cancer cells), HCT-116 (colon cancer cells), HL-60 (leukemia cells), and SF-295 (glioblastoma cells), was evaluated. All cell lines were donated by the National Cancer Institute (USA). Cytotoxicity was determined by the capacity of live cells to reduce the yellow dye 3-(4,5-dimethyl-2-thiazolyl)-2,5-diphenyl-2H-tetrazolium bromide (MTT) to an insoluble purple formazan product [32]. Cell lines were grown in RPMI 1640 medium (Gibco^®^, Life Technologies, Carlsbad, CA, USA) supplemented with 10% fetal bovine serum (Gibco^®^) and 1% penicillin at 37°C in the humidified atmosphere of 95% air/5% CO_2_. All cell lines were seeded at a concentration of 1×10^6^ cells/mL in 96-well plates. After 24 h, the extracts dissolved in 1% DMSO to a concentration of 50 μg·mL^-1^ were added to each well.

The plates were incubated for 72 h at 37°C in the atmosphere of 95% air/5% CO_2_. Cells treated with pure and sterile 1% DMSO (without propolis extracts) were used as a negative control (untreated cells). For positive control, cells were treated with 100 μg∙mL^-1^ doxorubicin (purity >98%; Sigma Chemical Co., St. Louis, MO, USA). Positive control cells were maintained under the same conditions as treated cells [33]. Then, the plates were centrifuged, and the medium was replaced by fresh medium (150 μL) containing 0.5 mg∙mL^-1^ MTT. The plates were then incubated for another 3 h. The absorbance was measured with a spectrophotometric plate reader (DTX 880 Multimode Detector, Beckman Coulter Inc.) at 595 nm. Median concentrations of propolis extracts able to induce half-maximal inhibitory effect (IC_50_) were then determined.

### Statistical analysis

Data are presented as the mean ± standard error of the mean or as half-maximal inhibitory concentration (IC_50_) values. The 95% confidence intervals were obtained through nonlinear regression. Statistical significance was evaluated using the one-way analysis of variance by Statistica^®^ 6.0 software (StatSoft, Tulsa, USA). The *post hoc* Tukey’s test was used to determine statistical significance of differences (*P* < 0.05) between the means of experimental groups.

## Results and discussion

### Antioxidant activity *in vitro*

**Fig 1:**
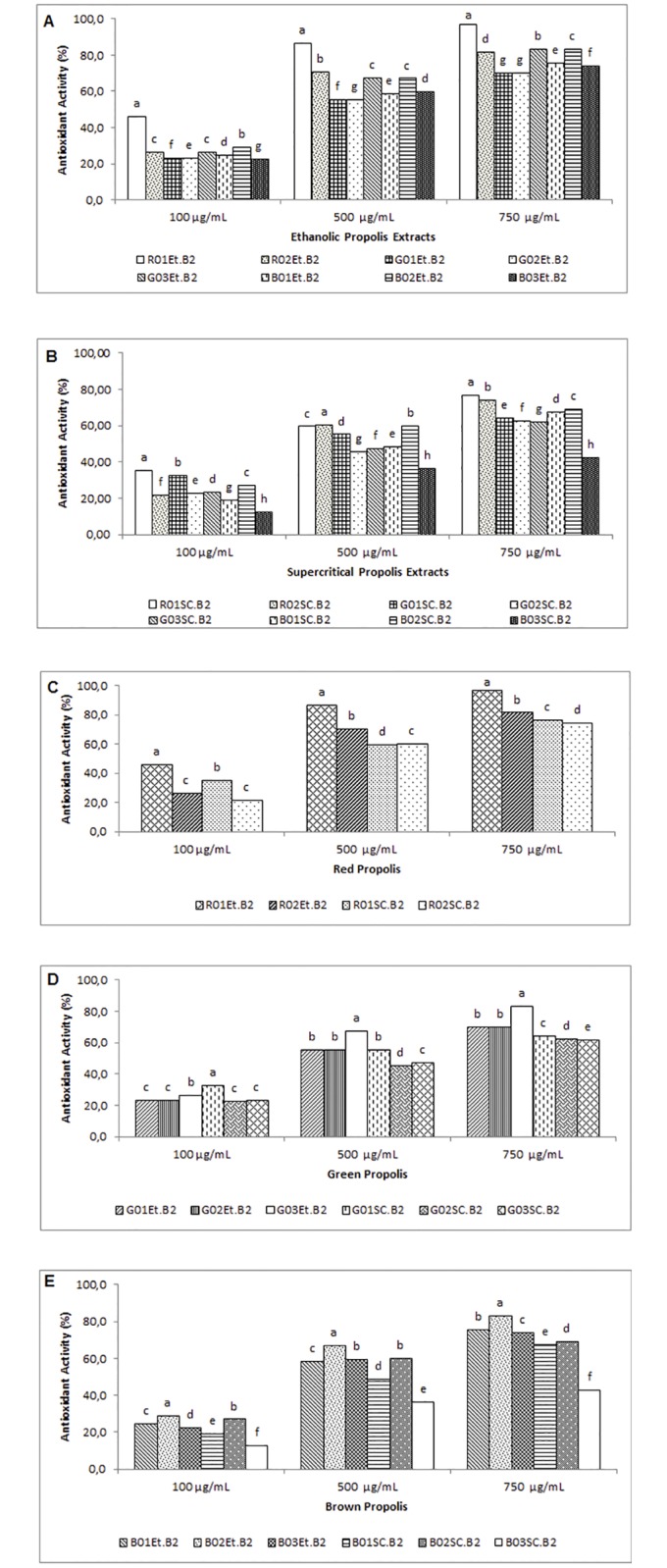
Determination of antioxidant activity of the propolis extracts from different regions of Brazil by the ABTS method, using four different concentrations (100, 500and 750 μm.mL^-1^), expressed as a percentage of antioxidant activity. Fig 1A –Extracts obtained by ethanolic extraction; Fig 1B –Extracts obtained by Supercritical extraction; Fig 1C –Comparison between red propolis extracts; Fig 1D—Comparison between green propolis extracts; Fig 1E—Comparison between brown propolis extracts. Values showing different letter on the same concentration for different propolis extracts show significant difference (p>0.05) through the Tukey test at 95% confidence level. Et—Extracts obtained by ethanolic extraction. SC—Extracts obtained by Supercritical extraction. Average of analysis obtained in triplicate (n = 3).

The antioxidant activity of ethanolic and supercritical extracts analyzed in this study is illustrated in Fig 1 (S1 and S2 Tables).

Propolis extracts showed antioxidant activity (expressed as a percentage of antioxidant activity) at three different concentrations (100, 500 and 750 μg∙mL^-1^). As shown in Fig 1, the antioxidant effects of ethanolic and supercritical propolis extracts were concentration-dependent.

All ethanolic extracts at a concentration of 500 μg∙mL^-1^ had high antioxidant activity of over 50%. Moreover, at two higher concentrations, ethanolic extracts showed antioxidant activity of over 70%. At a concentration of 100 μg∙mL^-1^, all extracts exhibited weak antioxidant activity (less than 50%). Ethanolic extract of red propolis R01Et.B2 showed strong antioxidant activity of more than 95% at a concentration of 750 μg∙mL^-1^. All supercritical extracts of propolis also exhibited strong antioxidant activity (over 60%) at that concentration, except for B03SC.B2, which had weak antioxidant activity.

Data from samples obtained by ethanolic and supercritical extraction methods were significantly different (*P* < 0.05) in the majority of samples analyzed (Fig 1C–1E). These results confirmed that extraction method significantly affected the chemical composition of the final extract formed.

In a previous study by our group [25], samples collected in the same geographic region were evaluated at a concentration of 1 mg∙mL^-1^ by the ABST method. As observed in this study, red propolis samples exhibited the highest antioxidant activity. Overall, the extracts obtained by ethanolic extraction had more pronounced effects than those obtained by supercritical extraction.

It is important to note that significant differences in efficacy were observed between the same samples tested at concentrations of 100, 500 and 750 μg∙mL-^1^, but extracted by different methods. We made a similar observation previously during the evaluation of the same samples at a concentration of 1000 μg∙mL^-1^ [25]. However, in our previous study, the highest antioxidant activity, as determined by the DPPH method (2,2-diphenyl-1-picrylhydrazyl), was observed with the ethanolic extract of green propolis from Minas Gerais (IC_50_ of 31.80 ± 0.16), with the ethanolic extract of red propolis from Alagoas being next most active preparation (IC_50_ of 44.29 ± 0.29) [25].

Notably, when comparing the results of the present study to those obtained by Machado et al. [25], it is evident that in relation to the antioxidant activity, propolis samples collected in the same geographic region demonstrated similar properties, although they were collected at different periods. In addition, we conclude that red propolis samples had the highest overall antioxidant potential, and that the conventional method of extraction by ethanol produced extracts with stronger antioxidant activity, irrespective of the type of sample evaluated (red, green, or brown propolis).

When analyzing multiple propolis samples collected in the same geographic areas of Europe, North America, Africa, and Brazil by electrospray ionization mass spectrometry, Sawaya *et al*. [4] observed that samples originating from the same area had similar characteristics, irrespective of the collection period [4]. However, concentrations of some constituents were affected by the collection period. In addition, for propolis samples from Brazil, the chemical composition was relatively diverse, which was attributed to the diversity of geographical regions of collection.

Valencia *et al*. [34] evaluated the effect of season on the chemical composition and biological activity (antiproliferative and antioxidant effects) of propolis from Mexico, and showed that the season of collection affected only antiproliferative properties.

Propolis antioxidant activity has been attributed to the high content of phenolic compounds and flavonoids in this natural substance [8, 9, 25, 35]. In our previous study, we showed that there was a positive correlation between the level of antioxidant activity and concentrations of phenolic compounds and flavonoids [25]. Moreover, ethanolic extracts showed a higher antioxidant activity than supercritical extracts, which may be explained by the fact that ethanol extraction yielded greater amounts of polyphenols and flavonoids, and consequently, enabled higher antioxidant capacity [25, 36, 37].

The results obtained in our experiments corroborate observations made in the studies by Anh *et al*. [8] and Choi *et al*. [9]. Those authors showed that antioxidant activity of propolis extracts from China and Korea, respectively, varied according to the exact region from which propolis samples were collected. Furthermore, other authors have described the influence of the extraction method on the antioxidant activity of propolis samples [22–24, 36, 37].

In Brazil, because of the country’s great biodiversity, it was expected that activities of the samples analyzed in this study would be different from each other (*P* < 0.05), because they were collected from different geographical regions.

### Antimicrobial activity

Antimicrobial and antifungal properties of propolis have been extensively studied with regard to their potential applications in pharmaceutical and food industries [11].

In this study, antimicrobial and antifungal activities of propolis were evaluated by determining MIC values during incubations of samples with different pathogens. Table 2 shows the results of *in vitro* antimicrobial activity testing of propolis extracts obtained by ethanolic and supercritical extraction.

**Table 2:** Determination of Minimal Inhibitory Concentration (MIC) of extracts from different samples of Brazilian propolis obtained by Ethanolic extraction (Et) and by Supercritical fluid extraction (SC). MIC is expressed as μg.mL^-1^.

Extracts	*Enterococcus sp*. ATCC 2912	*Staphylococcus aureus* ATCC 25923	*Klebsiella sp*. ATCC 1706/ 700603	*Escherichia coli* ATCC 25922	*Candida albicans* ATCC 1880
**R01Et.B2**	62.5	125	62.5	>1000	>1000
**R02Et.B2**	31.3	62.5	31.3	>1000	>1000
**G01Et.B2**	250	500	500	>1000	>1000
**G02Et.B2**	250	>1000	>1000	>1000	>1000
**G03Et.B2**	250	250	>1000	>1000	>1000
**B01Et.B2**	>1000	>1000	>1000	>1000	>1000
**B02Et.B2**	>1000	>1000	>1000	>1000	>1000
**B03Et.B2**	500	1000	>1000	>1000	>1000
**R01SC.B2**	125	250	250	>1000	>1000
**R02SC.B2**	62.5	125	62.5	>1000	>1000
**G01SC.B2**	>1000	250	>1000	>1000	>1000
**G02SC.B2**	>1000	500	>1000	>1000	>1000
**G03SC.B2**	>1000	250	>1000	>1000	>1000
**B01SC.B2**	>1000	>1000	>1000	>1000	>1000
**B02SC.B2**	>1000	>1000	>1000	>1000	>1000
**B03SC.B2**	>1000	>1000	>1000	>1000	>1000

Minimal inhibitory concentration (MIC) of the red, green, and brown propolis extracts obtained by ethanolic extraction (Et) and by supercritical fluid extraction (SC) at concentrations from 31.3 to 1000 μg.mL^-1^.

We observed that red propolis showed the highest antimicrobial activity among the samples obtained by both ethanolic and supercritical extraction methods. R02Et.B2 sample (ethanolic red extract from Alagoas) exhibited the highest antimicrobial activity against *Enterococcus* sp., *Staphylococcus aureus*, and *Klebsiella* sp. with MIC values of 31.3, 62.5, and 31.3 μg∙mL^-1^, respectively. Similar results were reported in other studies that also described strong antimicrobial activity of red propolis from Brazil [22, 29, 38]. Extracts of green propolis exhibited moderate to weak antimicrobial activity (MIC values ranging from 250 to 500 μg∙mL^-1^) for most samples, whereas extracts of brown propolis did not show any antimicrobial or antifungal activity against the bacterial strains and fungi tested.

None of the analyzed extracts was active against *E*. *coli* or *C*. *albicans*. A similar result was obtained by Popova *et al*. [39], who analyzed antimicrobial activity of Mediterranean propolis from Malta. Likewise, Bankova *et al*. [40] did not observe any inhibitory activity of extracts of Brazilian and Bulgarian propolis against *E*. *coli*. However, other authors have described antimicrobial activity of propolis extracts against *E*. *coli* and *C*. *albicans* [12, 13, 18, 25]. These discrepancies can be explained by different chemical properties of propolis extracts used in various studies as well as by different concentrations of extracts used in the assays.

The results of our study are in agreement with published data that indicated high antimicrobial activity of propolis against gram-positive bacteria and low antimicrobial activity of propolis against gram-negative bacteria [3, 41–46]. Tiveron *et al*. [46] recently showed that organic propolis extracts did not affect *E*. *coli* at the maximum concentration tested (>1.600 μg∙mL^-1^). Taking into account the fact that the cell wall of gram-negative bacteria is chemically more complex, we can conclude that extract concentrations were not sufficiently high to inhibit growth of these bacteria. At the same time, negative results in the test of antifungal activity were unexpected, because propolis has been previously described as a potent antifungal agent [40, 47–49].

In our previous study, we analyzed antimicrobial activity against *S*. *aureus* and *E*. *coli* of propolis samples collected in the same geographic regions as those used in the present study [25]. Whereas previously we observed a significant antimicrobial activity of both ethanolic and supercritical extracts of red propolis against *E*. *coli* strains [25], we could not confirm those results in our current experiments.

With regard to the antimicrobial activity against *S*. *aureus*, the red propolis samples demonstrated potent inhibitory properties in both studies, albeit at different concentrations (MIC values ranging from 25 to 200 μg∙mL^-1^). Furthermore, green propolis samples from both studies also showed comparable effective concentration ranges (MIC values ranging from 200 to 400 μg∙mL^-1^), except for sample G02Et.B2. In our present study, brown propolis samples did not show antimicrobial activity against *S*. *aureus*, whereas in the previous study [25], they produced weak to moderate inhibition of *S*. *aureus* growth.

Observed differences in antimicrobial activity levels may have seasonal nature. According to the study by Bankova *et al*. [50], the chemical composition of propolis can be influenced by different temperature zones in the regions of propolis collection by bees. Castro et al. [51] documented the influence of seasonality on antibacterial activity and phenolic composition of propolis in southeastern and northeastern Brazil.

It should be noted that the number of studies that evaluated the effect of season on the chemical composition and biological activity of propolis samples from the same geographic origin is limited. In addition, published reports contain many discrepancies concerning the effect of season on biological activity of propolis extracts.

In the present study, we confirmed our previous data [25] and observed that seasonality had an important influence on antimicrobial activity, whereas its effect on antioxidant properties was less pronounced. In contrast to our data, in several studies, Sforcin *et al*. [52–54] found no significant seasonal differences in antimicrobial and immunomodulatory properties of propolis.

Simões-Ambrosio *et al*. [55] evaluated the effect of season on propolis samples collected monthly during one year and found that seasonality did not significantly alter qualitative chemical composition of propolis, whereas quantitative chemical profiles varied considerably.

Different MIC values observed in experiments with the same strains of bacteria can be explained by different times and locations of sample collection. Such relationships have also been demonstrated in other studies [25, 46, 56, 57]. As reported by us previously [25] and confirmed in our present study, among the samples evaluated, the extracts of red propolis exhibited the highest antimicrobial activity indicating a promising biological potential of this type of propolis [22, 29, 38].

It has been reported that propolis antimicrobial activity (similarly to antioxidant effect), depends on the presence of specific propolis constituents, such as flavonoids, phenolic acids, and others [4, 58, 59].

Most extracts analyzed in this study demonstrated high antimicrobial activity against *S*. *aureus*, a food-related microorganism. Antimicrobial effects of propolis extracts against *S*. *aureus* have been reported also in other studies [9, 11, 39, 41]. Therefore, our present and previously published results of antimicrobial testing of red propolis extracts suggest that future studies should focus on fractionation and isolation of bioactive compounds responsible for propolis antimicrobial properties.

### *In vitro* activity of the ethanolic extracts against *T*. *cruzi* epimastigotes

Chagas disease, also known as American trypanosomiasis, is a deadly disease caused by the protozoan parasite *T*. *cruzi*. This parasite is widespread in Latin America and is transmitted to humans via feces of triatomine bugs or due to the consumption of contaminated food [60].

**Fig 2:**
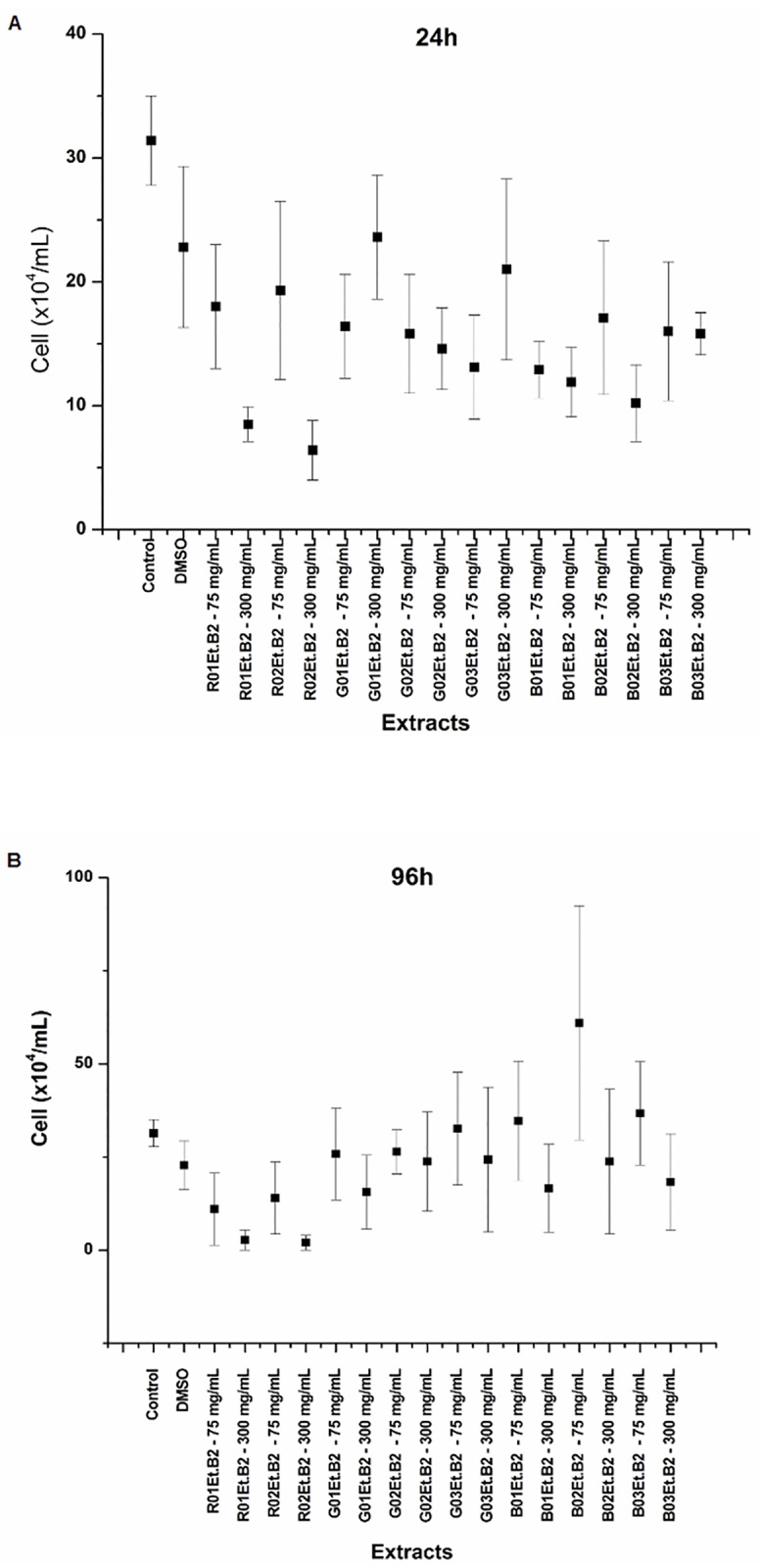
Activity of the EtOH extracts of different Brazilian propolis against *Trypanosoma cruzi* epimastigotes Y strains after 24 h (A) and 96 h (B) of incubation with both tested concentrations (75 and 300 mg.mL^-1^).

In this study, we investigated the effects of extracts of red, green, and brown propolis samples from Brazil on epimastigotes of *T*. *cruzi* Y strain, which is characterized by high pathogenicity, low parasitemia, and relatively high sensitivity to treatment [61]. Ethanolic extracts, which contained the highest levels of flavonoids and phenolic compounds according to our previous study, exhibited the strongest antioxidant activity. Thus, these extracts were chosen for *in vitro* evaluation of cytotoxic activity against *T*. *cruzi*. The results of these experiments are shown below (Fig 2 and S3 Table).

Ethanolic propolis extracts inhibited growth of *T*. *cruzi* epimastigote cultures at concentrations of 75 and 300 mg∙mL^-1^. In general, all samples analyzed in this study showed high inhibitory activity against *T*. *cruzi*, when compared to *T*. *cruzi* growth parameters in control conditions in the first 24 h. After 24 h, mean concentrations of epimastigotes were reduced by more than 90% by all extracts analyzed. The extract of red propolis R02Et.B2 showed the highest activity, leading to 98% inhibition of growth in 24 h of incubation.

However, in experiments with extracts of green and brown propolis (except for sample G01Et.B2 at a concentration of 300 mg∙mL^-1^), it was observed that the strength of epimastigote growth inhibition decreased in the interval from 24 h to 96 h. These results probably indicate uninhibited cell proliferation in the presence of extracts of green and brown propolis. This effect was also observed by Salomão *et al*. [17], when effects of different concentrations of green propolis against *T*. *cruzi* was analyzed. In that study, it was concluded that the inhibition of *T*. *cruzi* proliferation is dose-dependent. In contrast, inhibitory effect of the red propolis extract increased proportionally to the incubation time, indicating high biological potential of red propolis.

Ethanolic extracts of red propolis R01Et.B2 and R02Et.B2 samples exhibited the strongest inhibitory activity in comparison with the effects of other analyzed extracts. At a concentration of 300 mg.mL^-1^, R01Et.B2 and R02Et.B2 reduced concentrations of viable epimastigotes to 2.7 ± 0.7 and 2.0 ± 0.2 per mL after 96 h, respectively, which was equivalent to the reduction of 99%.

To the best of our knowledge, our experiments are the first to demonstrate biological activity of red propolis extracts against *T*. *cruzi*. Ayres *et al*. [62] reported that ethanolic extracts of Brazilian red propolis were the most active in reducing infections in macrophages of *Leishmania amazonensis*. The distinct chemical composition of red propolis [49, 63] may account for its higher biological activity.

It has been reported that the inhibition of the proliferation of *T*. *cruzi* epimastigotes and trypomastigotes could be proportional to the content of flavonoids and aromatic acids present in propolis extracts [16, 18,64]. This relationship can explain the superior activity of red propolis extracts compared to that of green or brown propolis samples analyzed, because red propolis has the highest content of flavonoids and phenolic compounds [25, 65]. This characteristic of red propolis extract was also confirmed by our group in a previous study that examined propolis samples of the same geographic origin [25].

Other *in vitro* studies showed that propolis inhibited growth of *T*. *cruzi* and of other pathogenic protozoans, such as *T*. *evansi*, *Giardia*, and *Leishmania* [49, 66–69]. However, Castro & Higashi [70] reported that mortality, parasitemia, or survival time of mice infected with *T*. *cruzi* was not affected when the mice were fed diets containing various formulations of propolis extract. Thus, further research on propolis activity in food formulations is necessary in order to develop a preparation that would demonstrate activity levels comparable to those seen in tests *in vitro*.

### Cytotoxicity *in vitro*

Propolis cytotoxic activity has been extensively studied, particularly in the area of cancer research [71–74]. In this study, *in vitro* cytotoxic activity of ethanolic extracts of propolis was investigated. We found that only samples of red propolis demonstrated potent cytotoxic activity against the tumor cell lines analyzed, whereas ethanolic extracts of green or brown propolis did not exhibit cytotoxic properties (data not shown). The results are shown in Table 3.

**Table 3:** *In vitro* cytotoxicity of the EtOH red extracts on tumor cell lines. Experiments were performed in triplicate.

Samples μg/mL	HL-60	HCT-116	OVCAR-8	SF-295
**Doxorubicin**	0.02	0.01	1.18	0.25
	0.01–0.02	0.01–0.03	0.92–1.51	0.16–0.35
**R01Et.B2**	4.80	19.92	23.63	13.67
	(3.97–5.82)	(14.40–27.56)	(19.66–28.40)	(11.22–16.65)
**R02Et.B2**	8.74	30.19	27.08	18.47
	(7.66–9.95)	(21.91–41.59)	(24.67–29.72)	(15.10–22.59)

Cell lines: OVCAR-8 (ovarian adenocarcinoma), HCT-116 (colon carcinoma), SF-295 (glioblastoma), and HL-60 (leukemia) humans. Data are presented as IC50 values (μg/mL), and their 95% confidence interval was obtained by non-linear regression from three independent experiments performed in triplicate, measured by the MTT assay after 72 h of incubation. Doxorubicin was used as the positive control.

The most pronounced cytotoxic effect of red propolis was against the leukemia (HL-60) tumor cell line, with IC_50_ values ranging from 3.97 to 5.82 μg∙mL^-1^ and between 7.66 and 9.95 μg∙mL^-1^ for R01Et.B2 and R02Et.B2 samples, respectively. R01Et.B2 sample (red propolis from Sergipe) was the strongest cytotoxic agent against all four cancer cell lines tested. These results indicate that red propolis extracts have potent cytotoxic properties. Novak *et al*. [73] reported IC_50_ values of 29.7 and 20.5 μg∙mL^-1^ for red propolis extract and its fraction, respectively. Carvalho *et al*. [75] reported IC_50_ values ranging between 25.67 and 33.72 μg∙mL^-1^ in experiments, where cytotoxicity of propolis from Paraná against HL-60 cells was examined.

R02Et.B2 and R01Et.B2 samples exhibited cytotoxic effects against glioblastoma (SF-295) tumor cell line with IC_50_ values of 11.22–16.65 μg.mL^-1^ and 15.10–22.59 μg∙mL^-1^, respectively. Cytotoxic activity of oil extracts of propolis against SF-295 tumors has been described previously [74, 75].

Colon tumor cells (HCT-116) were less sensitive to propolis extracts, with IC_50_ values ranging from 14.40 to 41.59 μg∙mL^-1^. Different results were observed by Buriol *et al*. [74], who showed that oil and ethanolic propolis extracts from Paraná-Brazil potently inhibited the proliferation of HCT-116 colon tumor cells. In our experiments with OVCAR-8 ovarian tumor cells, we found that as in the case with HCT-116 cells, the extracts of red propolis showed lower cytotoxic activity (IC_50_ values ranging from 19.66 to 29.72 μg∙mL^-1^) than in experiments with SF-295 and HL-60 cells.

Some constituents, such as polyisoprenylated benzophenone, xanthochymol, and isoflavone formononetin were associated with cytotoxic activity of red propolis when its fractions were tested separately [49, 63, 64]. However, other recent studies proposed that cytotoxic and anti-proliferative effects of propolis against tumor cells may not correlate exclusively with the concentration of a specific component, but instead rely on the synergism between actions of several components [73, 75].

The results described in the present study, as well as data from other studies that examined cytotoxic activity of propolis, underscore the importance of further evaluation of the actions of propolis extracts on different types of tumor cells, because propolis activity profiles may vary depending on the cell type assessed and propolis sample origin. Tests *in vivo* will be also required for the evaluation of possible side effects of propolis.

## Conclusions

This study demonstrated *in vitro* antioxidant, antimicrobial, antiparasitic, and cytotoxic properties of propolis extracts from Brazil. It was noted that ethanolic red propolis extracts had the highest activity in all performed tests. Growth of tumor cells was inhibited only by red propolis samples. In addition, red propolis displayed the highest activity against *T*. *cruzi* epimastigotes, caused greater inhibition of gram-positive bacteria, and exhibited higher antioxidant activity. The results of this study showed that propolis has a wide spectrum of biological activity, and that its properties depend on factors, such as the extraction method and region from which propolis was collected. Our present findings largely confirmed and significantly extended the results observed in other studies that examined biological effects of propolis extracts.

In summary, our data suggest the possibility of a wider usage of propolis extracts, especially those of red propolis that demonstrated the strongest biological activity. However, further investigations will be required to better delineate the conditions for inclusion of propolis in food formulations or its potential use in the pharmaceutical industry. New studies regarding the application of red propolis extracts in food products are currently underway in our group.

## Supporting information

S1 Table Results of antioxidant activity of the propolis samples. Extracts obtained by ethanolic extraction.
(DOCX)

S2 Table Results of antioxidant activity of the propolis samples. Extracts obtained by ethanolic extraction. Extracts obtained by Supercritical extraction.
(DOCX)

S3 Table Results of activity of the EtOH extracts against *Trypanosoma cruzi* epimastigotes Y strains after 24 h and 96 h of incubation, in 75 and 300 mg.mL^-1^ tested concentrations. (DOCX)
