# Supplementary material for: Antioxidant, antimicrobial, antiparasitic, and cytotoxic properties of various Brazilian propolis extracts
Source: PLoS One. 2017 Mar 30;12(3):e0172585. doi: 10.1371/journal.pone.0172585 (PMC5373518; doi:10.1371/journal.pone.0172585)
Supplement: S1 Table — Extracts obtained by ethanolic extraction. (DOCX) [file pone.0172585.s001.docx]

**Supporting Information**

**S1 Table S1** Results of antioxidant activity of the propolis samples. Extracts obtained by ethanolic extraction.

Values representing the same letter, on the same column, do not show significant differences (p>0.05) by the Tukey Test at 95% confidence.
