# Supplementary material for: Antioxidant, antimicrobial, antiparasitic, and cytotoxic properties of various Brazilian propolis extracts
Source: PLoS One. 2017 Mar 30;12(3):e0172585. doi: 10.1371/journal.pone.0172585 (PMC5373518; doi:10.1371/journal.pone.0172585)
Supplement: S2 Table — Extracts obtained by ethanolic extraction. Extracts obtained by Supercritical extraction. (DOCX) [file pone.0172585.s002.docx]

**Supporting Information**

**S2 Table.** Results of antioxidant activity of the propolis samples. Extracts obtained by ethanolic extraction. Extracts obtained by Supercritical extraction.

Values representing the same letter, on the same column, do not show significant differences (p>0.05) by the Tukey Test at 95% confidence.
