# Supplementary material for: Antioxidant, antimicrobial, antiparasitic, and cytotoxic properties of various Brazilian propolis extracts
Source: PLoS One. 2017 Mar 30;12(3):e0172585. doi: 10.1371/journal.pone.0172585 (PMC5373518; doi:10.1371/journal.pone.0172585)
Supplement: S3 Table — (DOCX) [file pone.0172585.s003.docx]

**Supporting Information**

**S3 Table.** Results of activity of the EtOH extracts against *Trypanosoma cruzi* epimastigotes Y strains after 24 h and 96 h of incubation, in 75 and 300 mg.mL^-1^ tested concentrations.
